# Supplementary material for: Resolving the full spectrum of human genome variation using Linked-Reads
Source: Genome Res. 2019 Apr;29(4):635–45. doi: 10.1101/gr.234443.118 (PMC6442396; doi:10.1101/gr.234443.118)
Supplement: Supplemental Material [file supp_gr.234443.118_Supplemental_longranger-2.2.zip › longranger-2.2.tar.bz2!/lr2.2-public/lib/stan/cmdstan/stan/doc/api/html/initialize__variable_8hpp_source.html]

Stan: src/stan/agrad/rev/matrix/initialize\_variable.hpp Source File


|  |  |
| --- | --- |
|  | Stan  2.5.0  probability, sampling & optimization |


- Main Page
- Related Pages
- Namespaces
- Classes
- Files

- File List
- File Members

All Classes Namespaces Files Functions Variables Typedefs Enumerations Enumerator Friends Macros Pages

- src
- stan
- agrad
- rev
- matrix

initialize\_variable.hpp

Go to the documentation of this file.

1 #ifndef STAN\_\_AGRAD\_\_REV\_\_MATRIX\_\_INITIALIZE\_VARIABLE\_HPP

2 #define STAN\_\_AGRAD\_\_REV\_\_MATRIX\_\_INITIALIZE\_VARIABLE\_HPP

3

4 #include <vector>

5 #include <stan/math/matrix/Eigen.hpp>

6 #include <stan/agrad/rev/var.hpp>

7

8 namespace stan {

9  namespace agrad {

10

15  inline void initialize\_variable(var& variable, const var& value) {

16  variable = value;

17  }

18

23  template <int R, int C>

24  inline void initialize\_variable(Eigen::Matrix<var,R,C>& matrix, const var& value) {

25  for (int i = 0; i < matrix.size(); ++i)

26  matrix(i) = value;

27  }

28

32  template <typename T>

33  inline void initialize\_variable(std::vector<T>& variables, const var& value) {

34  for (size\_t i = 0; i < variables.size(); ++i)

35  initialize\_variable(variables[i],value);

36  }

37

38  }

39 }

40

41 #endif

stan::agrad::initialize\_variable

void initialize\_variable(var &variable, const var &value)

Initialize variable to value.

**Definition:** initialize\_variable.hpp:15

var.hpp

Eigen.hpp

stan::agrad::var

Independent (input) and dependent (output) variables for gradients.

**Definition:** var.hpp:27


---

[ Stan Home Page ]

*© 2011–2014,
Stan Development Team.*
